# Volatile organic compounds in 169 energy‐efficient dwellings in Switzerland

**DOI:** 10.1111/ina.12667

**Published:** 2020-04-13

**Authors:** Shen Yang, Vincent Perret, Corinne Hager Jörin, Hélène Niculita‐Hirzel, Joëlle Goyette Pernot, Dusan Licina

**Affiliations:** ^1^ Human‐Oriented Built Environment Lab School of Architecture Civil and Environmental Engineering École Polytechnique Fédérale de Lausanne Lausanne Switzerland; ^2^ TOXpro SA Carouge Switzerland; ^3^ School of Engineering and Architecture of Fribourg HumanTech Institute HES‐SO University of Applied Sciences and Arts Western Switzerland Fribourg Switzerland; ^4^ Department of Health, Work and Environment Center for Primary Care and Public Health (Unisanté) University of Lausanne Lausanne Switzerland; ^5^ School of Engineering and Architecture of Fribourg Transform Institute Western Swiss Center for Indoor Air Quality and Radon (croqAIR) HES‐SO University of Applied Sciences and Arts Western Switzerland Fribourg Switzerland

**Keywords:** building characteristics, dwellings, energy efficiency, thermal retrofit, ventilation, volatile organic compounds

## Abstract

Exposure to elevated levels of certain volatile organic compounds (VOCs) in households has been linked to deleterious health effects. This study presents the first large‐scale investigation of VOC levels in 169 energy‐efficient dwellings in Switzerland. Through a combination of physical measurements and questionnaire surveys, we investigated the influence of diverse building characteristics on indoor VOCs. Among 74 detected compounds, carbonyls, alkanes, and alkenes were the most abundant. Median concentration levels of formaldehyde (14 μg/m^3^), TVOC (212 μg/m^3^), benzene (<0.1 μg/m^3^), and toluene (22 μg/m^3^) were below the upper exposure limits. Nonetheless, 90% and 50% of dwellings exceeded the chronic exposure limits for formaldehyde (9 μg/m^3^) and TVOC (200 μg/m^3^), respectively. There was a strong positive correlation among VOCs that likely originated from common sources. Dwellings built between 1950s and 1990s, and especially, those with attached garages had higher TVOC concentrations. Interior thermal retrofit of dwellings and absence of mechanical ventilation system were associated with elevated levels of formaldehyde, aromatics, and alkanes. Overall, energy‐renovated homes had higher levels of certain VOCs compared with newly built homes. The results suggest that energy efficiency measures in dwellings should be accompanied by actions to mitigate VOC exposures as to avoid adverse health outcomes.

**Practical implications**

- The results from this study provide a new large dataset on the individual VOC levels in energyefficient dwellings, which is valuable in relation to how exposure to VOCs influences human health.
- The levels of the most prevalent VOCs in Swiss dwellings are comparable to those in other European countries.
- Thermal retrofit of dwellings and absence of mechanical ventilation system are associated with elevated levels of formaldehyde, toluene and butane indoors.
- Energy‐efficiency measures in dwellings should be accompanied with actions to mitigate VOC exposures.
- The results are of potential utility for improving the indoor air quality models, for enhancing the ventilation design in energy‐efficient dwellings, and for improving the energy renovation processes.

## 1 INTRODUCTION

Volatile organic compounds (VOCs) are important gaseous pollutants, because many of them have known adverse effects on human health and comfort, ranging from mild irritation to acute toxicity and carcinogenicity. ^1^, ^2^, ^3^, ^4^ Indoors, VOCs are the most prevalent air pollutants and also the most studied. Lowering indoor VOC concentrations can improve working productivity and reduce health effects.^5^, ^6^ Indoor VOC concentrations are influenced by different factors, such as building characteristics, occupants’ behaviors, and environmental parameters.^7^, ^8^, ^9^, ^10^ Specifically in residential buildings where people spend 68% of their time,^11^ known sources of VOCs include their off‐gassing from building materials,^12^ paints,^13^ consumer and household products,^14^ occupants,^15^ secondary formation owing to indoor chemistry,^16^, ^17^ and intrusion of VOC‐enriched outdoor air.^18^ Owing to changes in residential materials, construction techniques and associated energy‐saving measures, increased use of consumer products, and altered occupants’ habits, the type and abundance of indoor VOCs have dramatically changed over the last decades.^19^ Understanding the VOC levels in residences is therefore important to better interpret their influence on occupants and to develop adequate control interventions.

Several studies provide abundant information on VOC contamination status in a sample of residences via population‐based investigation approach, as summarized by Logue et al,^20^ some of which investigated associations between dwelling characteristics and measured VOCs. Park and Ikeda measured 17 VOCs and 11 aldehydes in 1417 homes in Japan; they found that the VOC levels decrease with building age.^21^, ^22^ Raw et al^23^ reported a nationwide survey of 876 residences in England, in which concentrations of formaldehyde and TVOC (total VOC) were higher in newer than in older homes. Correlation between indoor formaldehyde and building age was also found in 100 dwellings in Hong Kong, while no such trend was witnessed for 15 other measured VOCs.^24^ The study in California investigated 24 VOCs in 108 newly built homes and found that formaldehyde concentrations were affected by ventilation type and geographical location of the dwellings.^25^ Similar conclusions were drawn in the measurement campaign in 305 dwellings in Sweden.^26^ The French Observatory for Indoor Air Quality (IOAQ) measured VOCs in 567 French homes, in which the influence of building characteristics and socioeconomic factors on VOCs was analyzed.^27^, ^28^ It was found that VOC levels are influenced by relative humidity, building age, garage type, and family wealth status. Cheng et al^29^ characterized indoor VOCs in 40 dwellings in Australia and found an association between proximity of major roads and indoor concentrations of alkanes and aromatics.

VOCs in energy‐efficient buildings are another area of increased public interest.^30^ Overwhelming focus on energy savings may often be in conflict with maintaining the recommended levels of indoor VOCs. The requirement for airtightness in energy‐efficient buildings can lead to low air infiltration, and if not sufficiently compensated by intentional ventilation, it can lead to higher VOC concentrations than that of conventional buildings.^31^ Thus, Langer et al^32^ reported higher TVOC concentrations in passive low‐energy houses compared with conventional ones in Sweden. Introduction of thermal retrofitting materials during residential energy renovation was found to contribute to elevated concentrations of indoor formaldehyde and TVOC.^33^ A recent field investigation by Du et al^34^ found that BTEX (benzene, toluene, ethylbenzene, and xylenes) concentrations significantly increased after energy renovation of multifamily dwellings in Finland. A field campaign in France showed that energy‐efficient houses had higher levels of several VOCs and aldehydes (acetaldehyde, hexaldehyde, n‐decane, n‐undecane, o‐xylene, and styrene) compared with the national average levels.^35^ In contrary, several studies reported reduced VOC levels in low‐energy houses compared with those conventionally built.^36^, ^37^ As summarized by Abadie and Wargocki^38^ through comparing the worldwide data of indoor VOC levels in conventional and low‐energy residences, energy‐efficient dwellings had higher indoor concentrations of alpha‐pinene, dodecane, and styrene, while levels of several other VOCs, such as toluene, were significantly lower in energy‐efficient residences. It was also pointed out that there is a need to build larger datasets of indoor VOCs for energy‐efficient dwellings. Other prominent VOC investigations in energy‐efficient residences have been reported by Kaunelienė et al^39^ and Derbez et al^40^ in Lithuania and France, respectively. Despite the reported findings, knowledge of the associations between energy‐efficient dwelling characteristics and VOC levels remains limited.^38^

Recently, Switzerland introduced the “Energy Strategy 2050” to reduce the energy‐related environmental impact.^41^ Key efforts have been made through construction of new energy‐efficient buildings and the promotion of a nationwide building energy renovation program (*Programme Bâtiment*).^42^ Overarching emphasis on energy‐saving measures raised important concerns about the associated VOC levels. To bridge this knowledge gap, we conducted the first large‐scale investigation of 169 newly built and renovated energy‐efficient dwellings in Switzerland. The objectives of this study were to (a) understand the indoor VOC contamination status in Swiss energy‐efficient homes using objective measurements and to compare the results with related campaigns reported in the literature; and (b) to probe the associations between measured VOCs and dwelling characteristics. Passive samplers for VOCs and aldehydes were applied for the field measurements. We used questionnaire surveys to collect information about building characteristics. This research contributes to better understanding of the indoor VOCs, enriching the scarce body of literature with VOC levels in energy‐efficient residences and potential improvement of energy renovation of buildings.

## 2 MATERIALS AND METHODS

### 2.1 Study design

Study samples were collected within the framework of the large‐scale survey conducted in “Mesqualair” New Regional Policy collaborative project on IAQ evaluation in 650 energy‐efficient dwellings from January 2013 to March 2016 in Western Switzerland.^43^, ^44^ 200 participants, largely building owners, were invited to take part in a complementary analysis of VOCs and aldehydes in their dwellings. A sampling kit for VOCs and aldehyde was sent by post to the 200 participating dwellings (detailed in Section 2.3). The occupants of the dwellings were instructed to install the passive samplers in their master bedroom during one week in September 2015. Out of 200 dwellings, occupants of 169 dwellings used the kit following the instructions and sent it back for laboratory analysis. Furthermore, they all fulfilled a self‐administrated questionnaire regarding building characteristics, potential pollutant sources, and their habits, which were sent back by post along with the samplers.

### 2.2 Study sample

The locations of the 169 investigated dwellings are graphically represented in Figure S1. Table 1 summarizes the information collected about building characteristics and occupant habits in 169 investigated dwellings, of which 168 were occupied by the owners. Most dwellings involved in this study were individual or semi‐detached houses. Over 72% of homes (124) were energy‐retrofitted, benefiting from the nationwide energy renovation project (*Programme Bâtiment*) in Switzerland. The remaining 45 dwellings were new homes already designed and built with energy‐efficient goals. The applied energy renovation strategies included thermal retrofit of roof, walls, and floors. About 40% of the energy‐renovated dwellings had their heating systems replaced. Out of 65 occupants that reported upgraded thermal insulation of the houses, 44 (68%) had their facade insulated from the interior side. Only 30% of dwellings were equipped with mechanical ventilation system. A large proportion (76%) of the investigated dwellings were built after 1950. Masonry building structure and detached garage were the most popular features of the dwellings. Indoor smoking habits of occupants were reported only in 6% of surveyed homes.

**TABLE 1:** A summary of the characteristics of the 169 dwellings sampled in this study. Responses of “I do not know” are excluded

Dwelling characteristics	Number of dwellings
Type	
Individual or semi‐detached house	144
Apartment	8
Other	17
Built year	
2000‐2015	46
1975‐1999	35
1950‐1974	47
1900‐1949	18
Before 1900	22
Energy efficiency status	
Energy‐renovated	124
Built energy‐efficient	45
Building structure	
Masonry	101
Wood	17
Mixed	42
Other	6
Thermal insulation during energy renovation	
Exterior	21
Interior	44
Garage type	
Attached	63
Detached	104
Mechanical ventilation	
Yes	57
No	109
Indoor smoking habits	
Yes	11
No	155

### 2.3 VOC and aldehyde quantification

The sampling kit consisted of two passive devices for VOCs and aldehydes (TOXpro SA, Switzerland) in compliance with ISO 16017‐2^45^ and ISO 16000‐4^46^ standards, respectively. Following step‐by‐step instructions, the occupants placed one passive badge sampler for VOCs (carbon molecular sieve, Anasorb 747) and one passive sampler for aldehydes (2,4‐dinitrophenylhydrazine impregnated silica gel) in the master bedroom of each sampled dwelling. The two samplers were placed between 1.0 m and 1.7 m above the ground, away from windows and any prominent VOC emission sources—including perfume, potpourri, and scented candles. The distance between the two samplers was larger than 0.3 m, to avoid cross‐contamination of the samplers, and less than 1.0 m to ensure the measurement in the same area of the bedroom. The VOC and aldehyde measurement lasted for seven days. During the sampling period, the occupants were asked to keep their living habits as usual, without touching or moving the samplers. The occupants had an option to phone the project team in the event that any questions had arisen.

The collected samplers were sent to laboratory (Advanced Chemical Sensors Co. Ltd, Florida, USA) where they were analyzed under ISO 17025^47^ accreditation scheme. Chemicals retained in the VOC passive samplers experienced solvent desorption with carbon disulfide as described by OSHA Method 7.^48^ Then, the extracted components were analyzed by gas chromatography with a Restek Rxi‐1 capillary column (dimethyl cyclosiloxane 60 m, 0.25 mm i.d, and 1.00 μm film thickness) coupled with one mass selective detector (GC‐MS, Shimadzu Model GC/QP‐2010) for identification and quantification following the Method TO‐15 from US Environment Protection Agency (EPA).^49^ The GC oven temperature program was started at 60°C, held for 6 minutes, then raised to 200°C at 10°C/min, and then held for 6 minutes. A calibration curve was generated for each substance with authentic standard samples of various concentrations from Aldrich Chemical Company. With the calibration curves, we obtained concentrations of individual VOCs. The VOC passive sampler together with the analyzing method was capable to detect and quantify 184 VOCs with a maximum total adsorbed mass of 35 mg, detailed in Supporting information. The VOCs in the samples were identified by screening the VOC list. Compared to Tenax TA sampling tubes, the passive sampler used in this study was able to better retain and quantify VOCs with small molecules (C3‐C6). Therefore, for better representatives of indoor air samples, we considered the total amount of compounds detected in the VOC passive sampler as TVOCs, of which the concentration was quantified as toluene‐equivalent, rather than sum of C6‐C16 VOCs as recommended by ISO 16000‐6.^50^ Aldehydes, including formaldehyde, acetaldehyde, acrolein, propionaldehyde, butyraldehyde, benzaldehyde, glutaraldehyde, and hexaldehyde, were detected and quantified after acetonitrile extraction by high‐performance liquid chromatography (HPLC). The HPLC used was a Waters Alliance 2695 Separation Module and 2487 Dual l Absorbance Detector (365 nm) with a C18 column (3.5 micron, 10 cm, Waters XBridge). The eluent was a mixture of acetonitrile and water (ratio 60:40). Each aldehyde was identified by the retention time in comparison with certified reference materials (CRM, Aldrich).

The measurement accuracy of the sampling and analysis of VOCs and aldehydes was within 25%. The limit of quantification (LOQ) was about 0.2–0.3 μg/m^3^ depending on the molecules. Only values > LOQ were reported. Compensation of air temperature and relative humidity of the sampled indoor environment was not applied. The laboratory experiments indicated that the relative humidity has no measurable effect on the analysis results. Increases in air temperature from 24°C up to 37°C were found to have less than a 10% effect, which was within the overall measurement error.

### 2.4 Statistical analyses

The statistical analyses were performed using SPSS 21 software and customized coding in MATLAB R2014 software. The concentrations of formaldehyde and logarithmical transformed TVOC were normally distributed (seen in Figure S2). Therefore, the parametric *t* test (number of categories *k* = 2) and analysis of variance (ANOVA) test (*k* > 2) were performed to test the relationship of the two variables with the dwelling characteristics. We also performed the nonparametric Wilcoxon Mann–Whitney U test (*k* = 2) and Kruskal‐Wallis test (*k* > 2) for investigating relations of other VOCs with the building characteristics. The Bonferroni correction was applied for multiple pair comparison to correct the level of significance of *P*‐values. The concentration values below the LOQ were replaced by LOQ/2. In addition, the effect size (*ES*) quantifies the difference among groups and thus was calculated in this study. For parametric test, Cohen's *d ES* was obtained, while for nonparametric test, the eta‐squared (*η* ^2^) *ES* was calculated and then converted to Cohen's *d ES* for consistent comparisons.^51^, ^52^ The *ES* larger than 0.2, 0.5, and 0.8 indicated small, medium, and large effects of the variables, respectively.^51^

## 3 RESULTS AND DISCUSSION

### 3.1 Descriptive VOC data

Table S1 summarizes the descriptive statistics of the detected VOCs in 169 Swiss dwellings. Overall, 74 VOCs were detected. Out of 7 VOC categories, carbonyls, alkanes, and alkenes were the most frequent groups of compounds. As much as 26% (19/74) of the screened VOCs were found in more than 50% of sampled homes (see Table 2). This proportion is lower than that observed in the Australia campaign^29^ (77 out of 97 VOCs (79%)), but higher than that observed in the Swedish campaign^26^ (11 out of 124 (9%)). In our study, formaldehyde, hexaldehyde, and toluene had the highest incidence—they were found in all the sampled dwellings, followed by ethanol, benzaldehyde, butane, and acrolein. Unlike our findings, Swedish dwellings had the highest reported incidence of benzene, 2‐ethyl‐1‐hexanol, and 1‐butanol.^26^

**TABLE 2:** Descriptive statistics and incidence (%) and concentration (μg/m^3^) of the 19 major detected VOCs (median > LOQ) in 169 dwellings

Category	Compound	P25	P50	P75	Max	Mean (SD)	GM (GSD)	%>LOQ
Alkanes and alkenes	Butane	4.1	15	45	509	54 (96)	12 (9.4)	91
	n‐Heptane	4.6	6.2	9.3	87	9.0 (10)	4.5 (5.3)	89
	2‐Methylbutane	4.7	15	39	741	49 (104)	7.8 (14)	82
	Isobutane	2.0	7.4	22	351	22 (41)	3.9 (12)	79
	n‐Pentane	<LOQ	3.6	13	238	15 (32)	1.5 (16)	63
Aromatics	Toluene	14	22	45	559	51 (79)	28 (2.6)	100
	Xylenes	<LOQ	3.2	16	269	22 (46)	1.4 (19)	60
Terpenes	D‐Limonene	5.5	9.4	16	91	14 (16)	3.4 (12)	75
	alpha‐Pinene	<LOQ	3.6	5.9	77	4.5 (7.4)	0.8 (11)	57
Carbonyls	Formaldehyde	11	14	18	50	14 (5.8)	13 (1.6)	100
	Hexaldehyde	5.1	6.9	10	41	8.6 (6.0)	7.2 (1.7)	100
	Benzaldehyde	0.8	1.0	1.2	22	1.1 (1.6)	0.9 (1.7)	98
	Acrolein	0.4	0.5	0.7	3.6	0.5 (0.4)	0.4 (2.2)	91
	Propionaldehyde	0.4	0.8	1.1	2.8	0.7 (0.4)	0.5 (2.7)	88
	Acetaldehyde	0.3	0.5	1.1	5.1	0.8 (0.9)	0.4 (3.2)	86
	Acetone	3.4	8.4	16	166	13 (19)	4.3 (7.9)	85
	Ethyl acetate	2.8	7.2	18	415	19 (44)	4.3 (9.6)	83
Other	Ethanol	49	95	198	4025	177 (358)	92 (3.3)	99
	Isopropyl alcohol	<LOQ	2.3	18	426	19 (44)	1.0 (22)	50
TVOC	–	121	212	439	2292	384 (450)	237 (2.6)	–

Abbreviations: GM, geometric mean; GSD, geometric standard deviation. TVOC concentration was toluene‐equivalent; LOQ, limit of quantification; Max, maximum; P25, 25th percentile; P50, median; P75, 75th percentile; SD, standard deviation.

The median concentrations of most frequently detected VOCs (>50%) were generally lower than 25 μg/m^3^. The median concentration of TVOC was 212 μg/m^3^, with mean ± standard deviation values of 384 ± 450 μg/m^3^. For VOCs of known health concern, formaldehyde (14 ± 5.8 μg/m^3^), benzene (3.1 ± 7.3 μg/m^3^), toluene (51 ± 79 μg/m^3^), and xylenes (22 ± 46 μg/m^3^) exhibited relatively low mean concentrations (detailed in Section 3.2). For VOCs known as potential indoor chemistry precursors, the concentrations of D‐limonene (14 ± 16 μg/m^3^) and alpha‐pinene (4.5 ± 7.4 μg/m^3^) were low as well. However, the maximum detected concentration of toluene and TVOC reached as high as 550 and 2200 μg/m^3^, respectively.

### 3.2 Summary comparisons of VOC data

To understand the status of indoor VOC contamination in Swiss dwellings, we benchmarked the measured VOC data against several established guideline values. We selected five guidelines for comparison, including the Swiss Federal Office of Public Health (FOPH), the World Health Organization (WHO), the Office of Environment Health Hazard Assessment (OEHHA, California EPA, USA), and French and German Indoor Air Quality Guidelines. The FOPH guideline provides benchmark values for Swiss local residences; the WHO guideline is available worldwide and offers reference values for other guidelines in individual countries; the OEHHA reports reference exposure limits of abundant chemicals, which are important referent values to evaluate occupants’ health, while the French and German guidelines are representatives of European ones, and the two countries share similar dwelling characteristics with Swiss dwellings.

**FIGURE 1:**
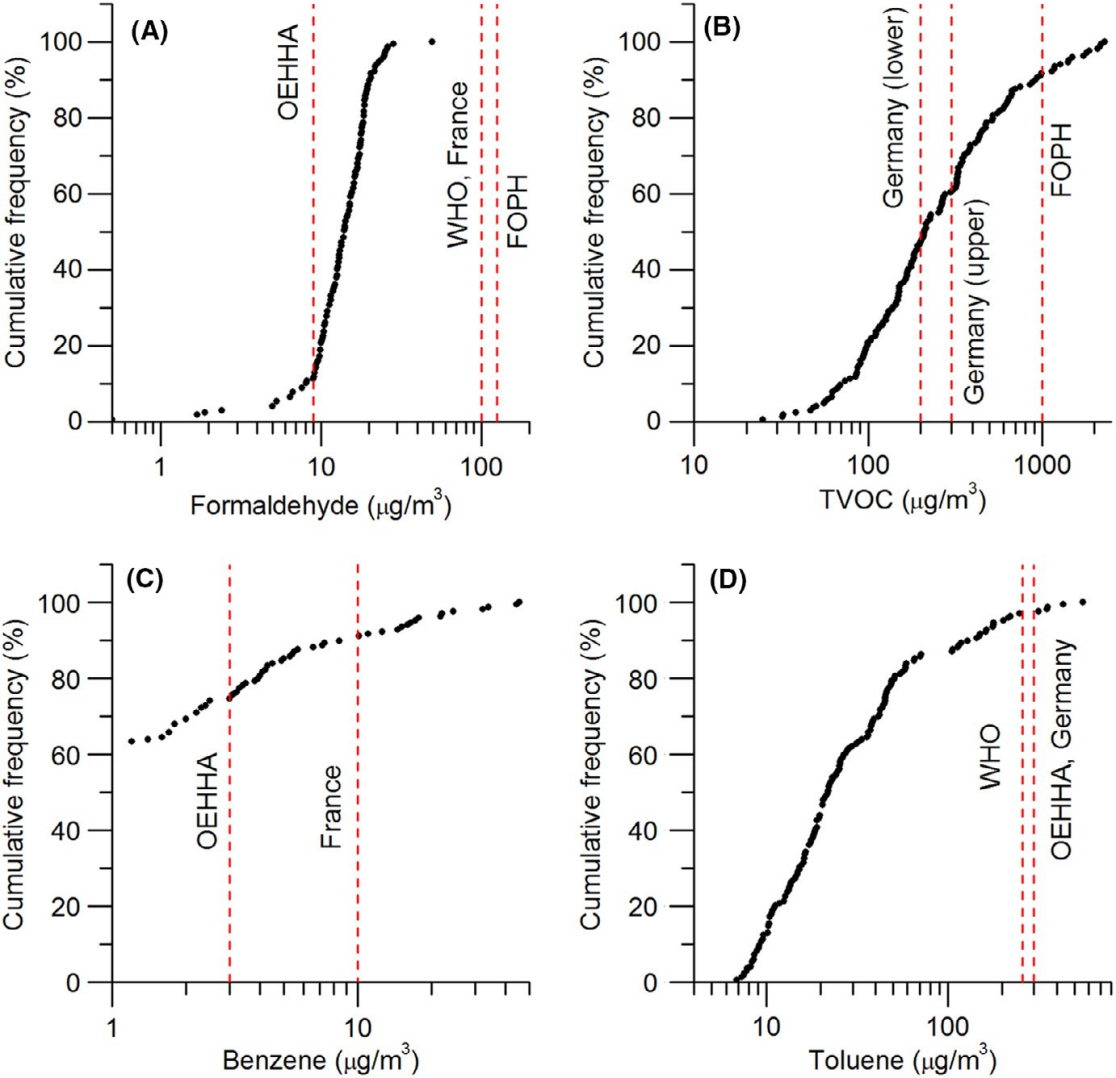
Cumulative frequency of concentrations of (A) formaldehyde, (B) TVOC, (C) benzene, and (D) toluene. Vertical dashed lines represent guideline values from OEHHA, WHO, FOPH, France, and Germany. The TVOC concentration was toluene‐equivalent

Figure 1 compares the cumulative frequency curves of formaldehyde, TVOC, benzene, and toluene against the referent values. For formaldehyde, the concentrations in all sampled dwellings were below the maximum recommended limit from the WHO^53^ and in France^54^ of 100 μg/m^3^ and from the FOPH of 125 μg/m^3^.^55^ Compared to the 8‐hour and chronic exposure limit value of 9 μg/m^3^ proposed by the OEHHA,^56^ the formaldehyde concentrations in as many as 90% of the sampled dwellings exceeded the threshold, which can lead to nasal obstruction and discomfort, lower airway discomfort, and eye irritation.^57^ For TVOCs, 8% of dwellings failed to stay below the Swiss upper exposure limit of 1000 μg/m^3^.^55^ The proportion of homes exceeding the TVOC limits was 53% and 40% when compared to the lower and upper 8‐hour exposure limit values from Germany (200 and 300 μg/m^3^, respectively).^58^ Benzene was detected in only 37% of sampled dwellings, among which 10% exceeded the French long‐term exposure guideline value of 10 μg/m^3^.^59^ Compared with OEHHA’s chronic exposure limit of 3 μg/m^3^,^60^ benzene concentrations were exceeded in over 25% homes—levels known to be associated with decreased peripheral blood cells.^61^ Considering the carcinogenicity, WHO recommends no safe level for exposure to benzene.^53^ Most of the sampled dwellings (>95%) were below the toluene concentration recommended by WHO (260 μg/m^3^),^53^ OEHHA,^62^ and Germany^58^ (300 μg/m^3^).

Table 3 compares the median concentrations of major detected VOCs in this study with the results from large field campaigns conducted in other countries. Formaldehyde concentrations in sampled Swiss dwellings corresponded to those detected in other European homes (most below 20 μg/m^3^ except in Lithuania), but below those measured in Hong Kong, Japan, and the United States. The concentrations of D‐limonene and TVOCs in this study were also comparable to those in other European countries. The Swiss homes had around two times higher indoor concentrations of toluene relative to other countries, while the benzene concentration was 1–3 μg/m^3^ higher.

**TABLE 3:** Comparison of median concentration of representative VOCs in Swiss residences with the related studies from other countries

Reference	No. of dwellings	Formaldehyde (μg/m^3^)	Benzene (μg/m^3^)	Toluene (μg/m^3^)	D‐Limonene (μg/m^3^)	TVOC (μg/m^3^)^d^
This work^a^	169	14	4.1	22	12	212
UK^23^ ^,^ ^c^	876	24	3.3	–	–	202
Japan^21^ ^,^ ^b^	1417	96	–	8.8	24	360
Hong Kong, China^24^ ^,^ ^b^	100	86	–	4.4	14	141
United States^25^ ^,^ ^c^	108	36	1.1	8.5	11	–
Sweden^26^ ^,^ ^b^	305	17	1.5	8.0	13	180
Sweden (energy‐efficient)^32^ ^,^ ^c^	20	11	0.8	3.7	4.5	272
Lithuania (energy‐efficient)^39^ ^,^ ^a^	11	31	0.8	4.1	–	–
Australia^29^ ^,^ ^c^	76	15	1.0	6.1	6.5	–
France^27^ ^,^ ^b^	576	20	2.0	12	–	–
France (energy‐efficient)^40^ ^,^ ^c^	72	19	0.7	3.4	13	–

^a^ Measurements were conducted in summer.

^b^ Measurements were conducted in winter.

^c^ Measurements were conducted in both summer and winter.

^d^ The range of VOCs included in TVOC could be different due to inconsistent sampling and analyzing methods.

Cross‐correlations among individual VOCs can to some extent reveal their potential emission sources. The matrix of Spearman coefficients for the 19 most frequently detected VOCs is presented in Table S2. The most frequently detected individual VOCs showed significant positive correlation with each other, though the coefficients were less than 0.50 in most cases. Notably, propionaldehyde, commonly found in ambient air released from manufacturing facilities, municipal waste incinerators, and combustions^63^ as well as from storage of wood pellets,^64^ exhibited negative correlation with most other compounds (*P* < .05), implying that the sources of propionaldehyde were different from other VOCs. Propionaldehyde may mainly originate from outdoors, while other compounds predominantly came from indoors, as suggested by the previously reported indoor/outdoor ratios in other studies.^29^ When the dwellings were ventilated via mechanical systems or opening the windows, the outdoor air introduced propionaldehyde to indoor environment but removed other indoor VOCs, leading to the negative correlations between the concentrations of propionaldehyde and other compounds. For aromatics (Table S3) and aliphatic compounds (Table S4), the Spearman correlation coefficients were much higher, at times > 0.90. The strong correlations indicate that the VOCs in different dwellings originate from the common sources. For instance, the major aromatic compounds are typically emitted simultaneously from solvents used for indoor decoration^65^ and from the air infiltration from the attached garage (described in Section 3.3).

### 3.3 Influence of dwelling characteristics on VOC concentrations

**FIGURE 2:**
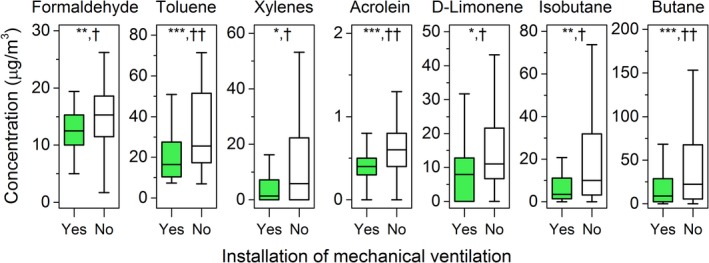
Comparisons of VOC concentrations according to the presence of the mechanical ventilation (yes, sample size: 57) or its absence (no, sample size: 109). *P < .05 = weakly significant, **P < .01 = significant, ***P < .001 = highly significant; †0.2 < effect size (*ES*) < 0.5 = small effect, ††0.5 < ES <0.8 = medium effect. Outliers were excluded from the figure

Relative to naturally ventilated residences, dwellings with installed mechanical ventilation systems had significantly lower median concentrations of several indoor VOCs, including formaldehyde (13 μg/m^3^ vs. 15 μg/m^3^), toluene (16 μg/m^3^ vs. 26 μg/m^3^), xylenes (1.4 μg/m^3^ vs. 5.8 μg/m^3^), acrolein (0.4 μg/m^3^ vs. 0.6 μg/m^3^), D‐limonene (7.9 μg/m^3^ vs. 11 μg/m^3^), isobutane (3.4 μg/m^3^ vs. 10 μg/m^3^), and butane (8.8 μg/m^3^ vs. 22 μg/m^3^), as shown in Figure 2. The similar findings that mechanically ventilated residences have lower VOC levels were reported in the French and Swedish campaigns.^26^, ^27^ Our previous study^44^ found that occupants in Swiss naturally ventilated dwellings tended to open window much less in winter than in summer. Thus, it can be assumed that in winter, the disparity in VOC levels between mechanically and naturally ventilated dwellings would be even larger, owing to reduced dilution of airborne contaminants in naturally ventilated homes.

**FIGURE 3:**
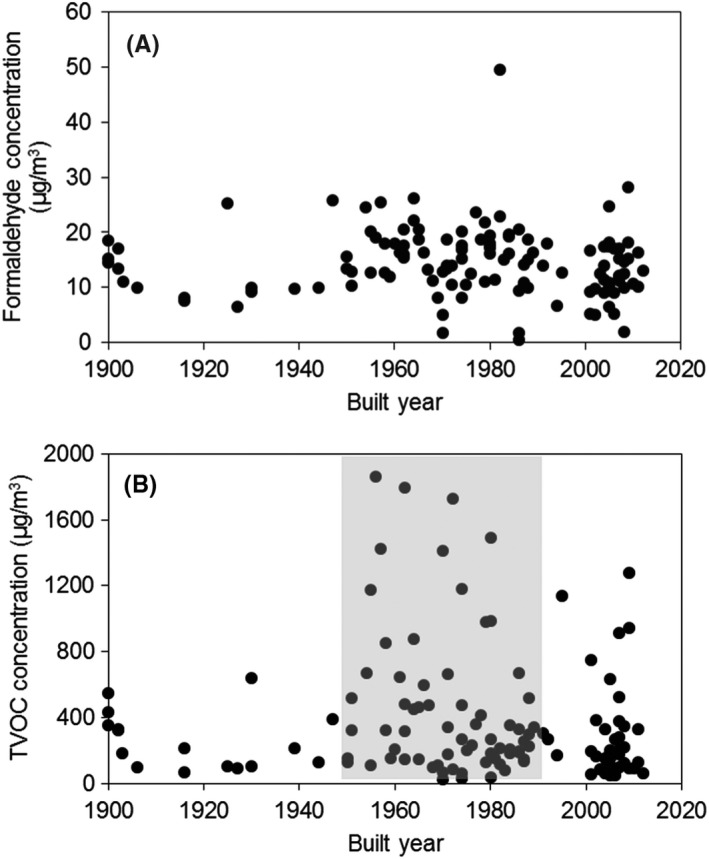
Relationship between the dwelling construction year and the concentration of (A) formaldehyde (*P *= .95) and (B) TVOC. Sample size: 145

Figure 3 shows the relationships between the dwelling construction year and the level of formaldehyde and TVOC. Interestingly, the concentrations of indoor formaldehyde did not vary with the building age (*P* = .95), which is inconsistent with the findings from the Swedish^26^ and French^27^ dwellings. This result can be attributed to the fact that majority of investigated dwellings (124/169) were energy‐renovated and had new sources of formaldehyde introduced during that process. On the other hand, the TVOC concentrations in dwellings built from 1950 to 1990 were significantly higher than the older and newer dwellings, which was in line with other European studies.^27^ The high TVOC concentrations may be a consequence of the absence of the mechanical ventilation system in dwellings built between 1950 and 1990 (Table S5) and low air infiltration rate.^26^

**FIGURE 4:**
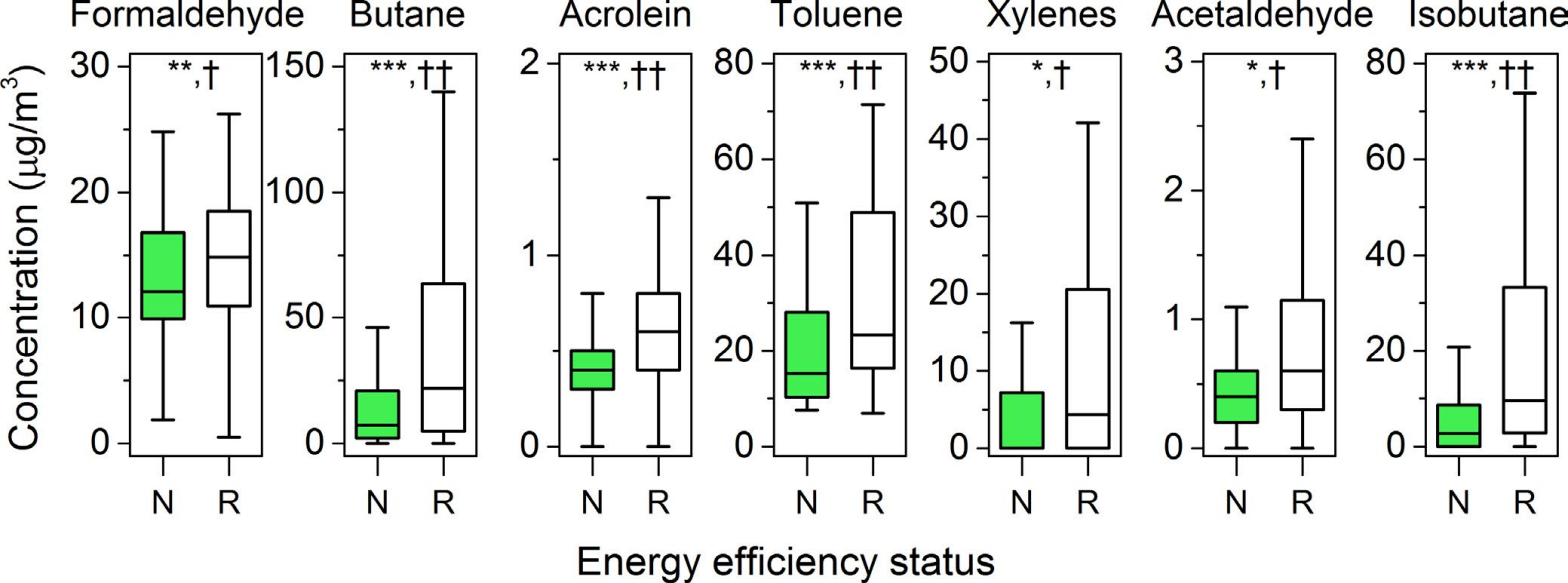
Comparisons of VOC concentrations according to energy efficiency status of sampled dwellings. N = newly built energy‐efficient, sample size: 45; *R* = energy‐renovated, sample size: 124. *P < .05 = weakly significant, **P < .01 = significant, ***P < .001 = highly significant; †0.2 < effect size (ES) < 0.5 = small effect, ††0.5 < ES <0.8 = medium effect. Outliers were excluded from the figure

Figure 4 shows that energy‐renovated dwellings had higher levels of certain VOCs compared with newly built ones. Specifically, median concentrations of formaldehyde (15 μg/m^3^ vs. 12 μg/m^3^), butane (22 μg/m^3^ vs. 7.4 μg/m^3^), acrolein (0.6 μg/m^3^ vs. 0.4 μg/m^3^), toluene (23 μg/m^3^ vs. 15 μg/m^3^), xylenes (4.4 μg/m^3^ vs. <LOQ), acetaldehyde (0.6 μg/m^3^ vs. 0.4 μg/m^3^), and isobutane (9.6 μg/m^3^ vs. 2.7 μg/m^3^) were significantly higher in energy‐renovated dwellings. The TVOC concentration was also higher in energy‐renovated dwellings than newly built ones (median 259 μg/m^3^ vs. 169 μg/m^3^) though without significance (*P* = .23). Because of the lack of data prior to renovation, we cannot associate the elevated VOC levels to thermal retrofitting. Nevertheless, the elevated VOC concentrations in renovated dwellings could be attributed to introduction of retrofitting materials into the houses during the renovation process and the absence of the mechanical ventilation, as only 13/124 renovated dwellings were equipped with the mechanical ventilation. On the contrary, all newly built energy‐efficient dwellings were mechanically ventilated. The influence of energy renovation can be further interpreted based on significant differences in several VOCs in dwellings with interior and exterior thermal insulation during retrofitting, as shown in Table S6. The median concentrations of formaldehyde (17 μg/m^3^ vs. 13 μg/m^3^), n‐heptane (8.1 μg/m^3^ vs. 5.4 μg/m^3^), xylenes (7.7 μg/m^3^ vs. <LOQ), and ethylbenzene (2.2 μg/m^3^ vs. <LOQ) were significantly higher in dwellings with interior thermal insulation, which can be attributed to VOC emissions from interior thermal insulation construction and materials.

Comparison between the two dwelling types (individual or semi‐detached house and apartment) did not reveal significant differences in VOC concentrations. Such findings differ from the results from the Swedish campaign^26^ where the concentrations of many detected VOCs were lower in apartments than in single‐family houses. The only significant difference (*P* < .01) was found for isobutane between the apartments (46 μg/m^3^) and the other houses (5.5 μg/m^3^). Nonetheless, the results should be interpreted with caution given the small number of sampled apartments (8).

We also probed the effects of dwelling material structure and garage type on the level of individual VOCs. Wood homes had significantly higher concentrations of several VOCs, that is, acrolein, toluene, glutaraldehyde, ethyl acetate, and 1‐butyl alcohol, compared to dwellings with masonry and mixed structures (Table S7), as similarly reported in the literature.^27^ Glutaraldehyde is widely used as the modification chemical to control wool moisture,^66^ which contributes to the higher concentrations in buildings of wooden structure. However, given the low sample size of wooden buildings (17), the results should be interpreted with care. Houses with attached garages had higher concentrations of formaldehyde, aromatics, and alkanes compared to those with detached garages (Table S8). The aromatics and alkanes are commonly associated with emissions from vehicles in garages.^67^ The infiltration of the VOCs from the attached garages to the living spaces contributed to the higher concentrations in the sampled bedrooms.

### 3.4 Study limitations

In interpreting the study results, a few limitations should be acknowledged. The measurements of VOCs were implemented by the occupants themselves, which may introduce some bias to the results. To minimize the potential influence of the self‐administered measurements on reliability and reproducibility of the results, we offered a comprehensive instruction to participants to cover factors including location and manipulation of the samplers, conditions of the sampling and return of the samplers. Comparable results with other European campaigns suggest a sufficient robustness of our dataset. Furthermore, we obtained the information about the ventilation type and occupants’ ventilation habits via questionnaire survey,^44^ but the ventilation rates during the measurements were unknown. This leads to a lack of quantitative association between ventilation rates and indoor VOC levels. The VOCs were quantified only during transition from summer season to autumn. Since the majority of sampled dwellings were naturally ventilated during the sampling period, the air exchange rates were likely higher than what would be during the winter (heating) season. Therefore, our results may underestimate the average VOC levels during the heating season. In addition, most of the dwellings involved in this study were single houses rather than apartment buildings. As building characteristics of apartments are usually different from houses, VOC concentrations in apartments are expected to vary from those in single houses. Further measurements in apartments are needed to understand the VOC levels in all types of energy‐efficient residential buildings in Switzerland.

## 4 CONCLUSIONS

This study presents the first large‐scale investigation of VOC levels in energy‐renovated and newly built energy‐efficient dwellings in Switzerland. The levels of the most prevalent individual VOCs were comparable to those in other European countries and were generally below the upper exposure thresholds. Nonetheless, the chronic exposure limits for formaldehyde (9 μg/m^3^) and TVOCs (200 μg/m^3^) were exceeded by over 90% and 50% of dwellings, respectively. Our results also reveal that different dwelling characteristics play a role in the accumulation of indoor pollutants. Dwellings built between 1950s and 1990s had higher TVOC concentrations than other periods. Energy renovation and the absence of mechanical ventilation were associated with higher indoor levels of formaldehyde, toluene, and butane. Attached garages contributed to higher indoor concentrations of formaldehyde, aromatics, and alkanes.

The results presented suggest that energy‐efficiency measures without consideration to Indoor Air Quality can compromise the level of VOCs. Efforts to construct new low‐energy homes and to upgrade existing ones to be more airtight and energy‐efficient should be accompanied by measures to secure adequate ventilation and to avoid introduction of high‐emitting materials. In summary, the presented results can be useful for verifying the compliance with existing guideline values for VOCs as well as for improving the Indoor Air Quality models, for enhancing the ventilation design and energy renovation procedures in energy‐efficient dwellings.

## CONFLICT OF INTEREST

None.

## Supporting information

Supplementary Material
