# Supplementary material for: Volatile organic compounds in 169 energy‐efficient dwellings in Switzerland
Source: Indoor Air. 2020 Apr 13;30(3):481–91. doi: 10.1111/ina.12667 (PMC7216845; doi:10.1111/ina.12667)
Supplement: Supplementary file 1 — Supplementary Material [file INA-30-481-s001.pdf]

Supporting information

Volatile organic compounds in 169 energy-efficient dwellings in Switzerland

Shen Yang [1], Vincent Perret [2,*], Corinne Hager Jörin [3], Hélène Niculita-Hirzel [4], Joëlle Goyette Pernot [5], Dusan Licina [1]

[1] Human-Oriented Built Environment Lab, School of Architecture, Civil and Environmental Engineering, École Polytechnique Fédérale de Lausanne, CH-1015 Lausanne, Switzerland

[2] TOXpro SA, CH-1227 Carouge, Switzerland

[3] HumanTech Institute, School of Engineering and Architecture of Fribourg, HES-SO University of Applied Sciences and Arts Western Switzerland, CH-1700 Fribourg, Switzerland

[4] Department of Health, Work and Environment, Center for Primary Care and Public Health (Unisanté), University of Lausanne, CH-1066 Epalinges, Switzerland

[5] Transform Institute, Western Swiss Center for Indoor Air Quality and Radon (croqAIR), School of Engineering and Architecture of Fribourg, HES-SO University of Applied Sciences and Arts Western Switzerland, CH-1700 Fribourg, Switzerland

*Corresponding author

Vincent Perret

TOXpro SA, CH-1227 Carouge, Switzerland

Phone: +41 76 581 04 65;

E-mail: vincent.perret@toxpro.ch

Table S1. Full table of descriptive statistics and incidence (%) and concentrations (μg/m^3) of the 74 detected VOCs in 169 dwellings.

Category	Compound	P25	P50	P75	Max	Mean (SD)	GM (GSD)	%> LOQ
Alkanes and alkenes	Butane	4.1	15	45	509	54 (96)	12 (9.4)	91
	n-Heptane	4.6	6.2	9.3	87	9.0 (10)	4.5 (5.3)	89
	2-Methylbutane	4.7	15	39	741	49 (104)	7.8 (14)	82
	Isobutane	2.0	7.4	22	351	22 (41)	3.9 (12)	79
	n-Pentane	<LOQ	3.6	13	238	15 (32)	1.5 (16)	63
	2-Methylpentane	<LOQ	<LOQ	9.6	393	14 (38)	0.7 (18)	49
	3-Methylhexane	<LOQ	<LOQ	4.3	138	5.4 (14)	0.2 (13)	34
	2-Methylhexane	<LOQ	<LOQ	4.2	122	5.3 (14)	0.2 (13)	34
	3-Methylpentane	<LOQ	<LOQ	4.2	185	5.7 (17)	0.2 (13)	32
	n-Hexane	<LOQ	<LOQ	<LOQ	107	4.4 (12)	<LOQ	24
	Isooctane	<LOD	<LOQ	<LOQ	84	2.8 (9.8)	<LOQ	21
	2,3-Dimethylpentane	<LOQ	<LOQ	<LOQ	16	0.6 (2.3)	<LOQ	9
	2-Methylheptane	<LOQ	<LOQ	<LOQ	10	0.3 (1.5)	<LOQ	7
	2,5-Dimethylhexane	<LOQ	<LOQ	<LOQ	17	0.3 (1.8)	<LOQ	6
	3,3-Dimethylpentane	<LOQ	<LOQ	<LOQ	3.0	<LOQ	<LOQ	2
	2-Methyl-1-Propene	<LOQ	<LOQ	<LOQ	11	<LOQ	<LOQ	1
Aromatics	Toluene	14	22	45	559	51 (79)	28 (2.6)	100
	Xylenes	<LOQ	3.2	16	269	22 (46)	1.4 (19)	60
	Ethylbenzene	<LOQ	<LOQ	3.5	51	3.8 (8.3)	0.3 (11)	42
	Benzene	<LOQ	<LOQ	3.1	46	3.1 (7.3)	0.2 (10)	37
	1,2,3-Trimethylbenzene	<LOQ	<LOQ	<LOQ	134	3.1 (12)	<LOQ	22
	2-Ethytoluene	<LOQ	<LOQ	<LOQ	179	5.0 (17)	<LOQ	18
	Propylbenzene	<LOQ	<LOQ	<LOQ	23	0.5 (2.1)	<LOQ	15
	4-Isopropyltoluene	<LOQ	<LOQ	<LOQ	46	0.7 (3.8)	<LOQ	13
	Isopropylbenzene	<LOQ	<LOQ	<LOQ	3.5	<LOQ	<LOQ	1
Cycloalkanes	Cyclopentane	<LOQ	<LOQ	4.9	107	4.8 (12)	0.3 (12)	40
	Methylcyclopentane	<LOQ	<LOQ	2.8	54	2.7 (7.4)	<LOQ	28
	Cyclohexane	<LOQ	<LOQ	<LOQ	44	1.6 (5.2)	<LOQ	18
	Methylcyclohexane	<LOQ	<LOQ	<LOQ	22	1.2 (3.1)	<LOQ	17
	1,2-Dimethylcyclopentane	<LOQ	<LOQ	<LOQ	14	<LOQ	<LOQ	2
	1,3-Dimethylcyclopentane	<LOQ	<LOQ	<LOQ	3.8	<LOQ	<LOQ	1
Terpenes	D-Limonene	5.5	9.4	16	91	14 (16)	3.4 (12)	75
	alpha-Pinene	<LOQ	3.6	5.9	77	4.5 (7.4)	0.8 (11)	57
	beta-Pinene	<LOQ	<LOQ	<LOQ	10	0.2 (1.2)	<LOQ	5
	Camphene	<LOQ	<LOQ	<LOQ	19	<LOQ	<LOQ	1
Carbonyls	Formaldehyde	11	14	18	50	14 (5.8)	13 (1.6)	100
	Hexaldehyde	5.1	6.9	10	41	8.6 (6.0)	7.2 (1.7)	100

	Benzaldehyde	0.8	1.0	1.2	22	1.1 (1.6)	0.9 (1.7)	98
	Acrolein	0.4	0.5	0.7	3.6	0.5 (0.4)	0.4 (2.2)	91
	Propionaldehyde	0.4	0.8	1.1	2.8	0.7 (0.4)	0.5 (2.7)	88
	Acetaldehyde	0.3	0.5	1.1	5.1	0.8 (0.9)	0.4 (3.2)	86
	Acetone	3.4	8.4	16	166	13 (19)	4.3 (7.9)	85
	Ethyl acetate	2.8	7.2	18	415	19 (44)	4.3 (9.6)	83
	Glutaraldehyde	<LOQ	<LOQ	0.6	5.9	0.4 (0.8)	0.1 (4.1)	50
	Butyl acetate	<LOQ	<LOQ	3.7	196	6.1 (21)	0.3 (12)	40
	2-Butanone	<LOQ	<LOQ	2.2	19	1.3 (2.7)	0.2 (7.4)	37
	Methyl acetate	<LOQ	<LOQ	<LOQ	21	1.1 (2.9)	<LOQ	22
	Butyraldehyde	<LOQ	<LOQ	<LOQ	6.4	<LOQ	<LOQ	20
	4-Methylpentan-2-one	<LOQ	<LOQ	<LOQ	16	0.4 (1.9)	<LOQ	7
	Propylene-glycol-methyl-ether-acetate	<LOQ	<LOQ	<LOQ	252	1.8 (19)	<LOQ	5
	Isoamyl acetate	<LOQ	<LOQ	<LOQ	42	0.5 (3.5)	<LOQ	5
	o-Phthalaldehyde	<LOQ	<LOQ	<LOQ	1.1	<LOQ	<LOQ	3
	Isobutyl acetate	<LOQ	<LOQ	<LOQ	12	<LOQ	<LOQ	1
	Isopropyl acetate	<LOQ	<LOQ	<LOQ	5.8	<LOQ	<LOQ	1
	n-Hexyl acetate	<LOQ	<LOQ	<LOQ	4.8	<LOQ	<LOQ	1
	2-Methylbutyl acetate	<LOQ	<LOQ	<LOQ	5.7	<LOQ	<LOQ	1
	Ethyl 2- methylbutyrate	<LOQ	<LOQ	<LOQ	6.1	<LOQ	<LOQ	1
Halocarbon	Isopropyl chloride	<LOQ	<LOQ	<LOQ	183	5.6 (24)	<LOQ	9
	Trichloroethylene	<LOQ	<LOQ	<LOQ	25	0.3 (2.7)	<LOQ	2
	Methylene chloride	<LOQ	<LOQ	<LOQ	114	0.8 (8.9)	<LOQ	1
	Perchloroethylene	<LOQ	<LOQ	<LOQ	371	2.1 (28)	<LOQ	1
Other	Ethanol	49	95	198	4025	177 (358)	92 (3.3)	99
	Isopropyl alcohol	<LOQ	2.3	18	426	19 (44)	1.0 (22)	50
	Methyl tert-butyl ether	<LOQ	<LOQ	<LOQ	79	4.3 (12)	<LOQ	25
	Dimethyl ether	<LOQ	<LOQ	<LOQ	66	1.2 (6.4)	<LOQ	9
	1,8-Cineole	<LOQ	<LOQ	<LOQ	55	2.0 (7.9)	<LOQ	9
	1-Butylalcohol	<LOQ	<LOQ	<LOQ	26	0.8 (3.3)	<LOQ	8
	Isobutanol	<LOQ	<LOQ	<LOQ	32	0.4 (2.6)	<LOQ	6
	Dimethoxymethane	<LOQ	<LOQ	<LOQ	11	<LOQ	<LOQ	2
	Hexamethyldisiloxane	<LOQ	<LOQ	<LOQ	13	<LOQ	<LOQ	1
	Ethyl ether	<LOQ	<LOQ	<LOQ	172	1.0 (13)	<LOQ	1
	Linalol	<LOQ	<LOQ	<LOQ	3.8	<LOQ	<LOQ	1
	Tetrahydrofuran	<LOQ	<LOQ	<LOQ	29	<LOQ	<LOQ	1
	1-Butoxy-2-propanol	<LOQ	<LOQ	<LOQ	4.3	<LOQ	<LOQ	1
TVOC	--	121	212	439	2292	384 (450)	237 (2.6)	--

Table S2. Spearman coefficient matrix of 19 most detected VOCs (>50%)

	C1	C2	C3	C4	C5	C6	C7	C8	C9	C10	C11	C12	C13	C14	C15	C16	C17	C18	C19
C1	1																		
C2	0.12	1																	
C3	0.30**	0.07	1																
C4	0.33**	0.32**	0.37**	1															
C5	0.55**	0.16*	0.32**	0.15*	1														
C6	-0.21**	0.03	-0.19*	-0.27**	-0.19*	1													
C7	0.75**	0.20**	0.33**	0.26**	0.73**	-0.25**	1												
C8	0.23**	0.22**	0.33**	0.30**	0.19*	-0.25**	0.28**	1											
C9	0.48**	0.26**	0.26**	0.15	0.50**	-0.12	0.53**	0.21**	1										
C10	0.26**	0.14	0.27**	0.47**	0.22**	-0.52**	0.30**	0.31**	0.25**	1									
C11	0.45**	0.19*	0.32**	0.47**	0.32**	-0.35**	0.43**	0.50**	0.30**	0.32**	1								
C12	0.12	0.03	0.34**	0.35**	0.11	-0.22**	0.12	0.39**	0.07	0.29**	0.42**	1							
C13	0.17*	0.51**	0.22**	0.57**	0.19*	0.01	0.15*	0.20**	0.07	0.23**	0.34**	0.17*	1						
C14	0.07	0.08	0.11	0.18*	-0.03	0.08	-0.04	0.07	0.08	0.06	0.08	0.28**	0.13	1					
C15	0.02	-0.02	0.02	0.13	0.11	-0.17*	0.06	0.00	0.00	0.00	0.15*	0.29**	0.11	0.12	1				
C16	0.15*	-0.01	0.01	0.19*	0.01	0.50**	0.04	-0.13	-0.06	-0.26**	0.06	0.06	0.20**	0.12	-0.09	1			
C17	0.23**	0.11	0.81**	0.36**	0.27**	-0.10	0.22**	0.28**	0.24**	0.25**	0.32**	0.36**	0.22**	0.12	0.03	0.07	1		
C18	0.31**	0.30**	0.30**	0.36**	0.29**	-0.24**	0.28**	0.36**	0.29**	0.19*	0.56**	0.27**	0.41**	0.04	0.19*	0.00	0.28**	1	
C19	0.56**	0.17*	0.39**	0.13	0.68**	-0.29**	0.68**	0.22**	0.40**	0.28**	0.33**	0.06	0.10	-0.22**	0.00	-0.04	0.33**	0.26**	1

C1 : Toluene, C2 : Benzaldehyde, C3 : Butane, C4 : Acrolein, C5 : n-Pentane, C6 : Propionaldehyde, C7 : Xylene,C8 : Ethyl acetate, C9 : n-Heptane, C10 :

Formaldehyde, C11 : D-limonene, C12 : Ethanol, C13 : Hexaldehyde, C14 : Isopropyl alcohol, C15 : Acetone, C16 : Acetaldehyde, C17 : Isobutane, C18 : AlphaPinene, C19 : 2-Methylbutane
**p*<0.05 ; ***p*<0.01

Table S3. Spearman coefficients across major aromatics

	Ethyl.Benzene	Propyl.Benzene	Toluene	Xylene	1,2,3-Trimethylbenzene	2-Ethytoluene	Benzene
Ethyl.Benzene	**1.00**						
Propyl. Benzene	0.69 **	**1.00**					
Toluene	0.74 **	0.60 **	**1.00**				
Xylene	**0.90** **	0.64 **	0.74 **	**1.00**			
1,2,3-Trimethylbenzene	0.74 **	**0.83** **	0.59 **	0.68 **	**1.00**		
2-Ethytoluene	0.72 **	**0.95** **	0.62 **	0.66 **	**0.81** **	**1.00**	
Benzene	**0.86** **	0.71 **	0.71 **	**0.83** **	0.75 **	0.72 **	**1.00**

Bold numbers represent Spearman coefficient larger than 0.80. ***p*<0.01

Table S4. Spearman coefficients across major aliphatic compounds

	2-Methylpentane	n.Hexane	3-Methylhexane	2-Methylhexane	3-Methylpentane	Methylcyclopentane
2-Methylpentane	**1.00**					
n-Hexane	0.77 **	**1.00**				
3-Methylhexane	0.72 **	0.80 **	**1.00**			
2-Methylhexane	0.74 **	**0.82****	**0.99** **	**1.00**		
3-Methylpentane	**0.88** **	**0.87** **	0.78 **	**0.80** **	**1.00**	
Methylcyclopentane	**0.80** **	**0.89** **	0.79 **	**0.81** **	**0.90** **	**1.00**

Bold numbers represent Spearman coefficient larger than 0.80. ***p*<0.01

Table S5. Installation of mechanical ventilation systems in dwellings of different ages, $p<0.001$

Built year	Mechanical ventilation system (No.)	
	Yes	No
2000-2015	45	0
1975-1999	2	32
1950-1974	2	44
1900-1949	4	14
Before 1900	4	18

Table S6. VOCs of significant difference between interior and exterior thermal insulation (median values, μg/m^3)

	Sample size	Ethylbenzene*,††	Xylene*,††	n-Heptane*,††	Formaldehyde**,††
Exterior	21	<LOD	<LOD	5.4	13
Interior	44	2.2	7.7	8.1	17

*$p<0.05$=weakly significant ,**$p<0.01$=significant ;††0.5<ES<0.8=medium effect

Table S7. VOCs of significant difference among building structures (median values, μg/m^3)

	Sample size	Acrolein*,†	Toluene**,†	Glutaraldehyde**,††	Ethyl Acetate**,††	1-Butylalcohol**,††
Masonry	101	0.5	23	LOD	9.3	<LOD
Wood	17	0.7	57	1.0	24	2.9
Mixed	42	0.6	19	0.4	5.5	<LOD

*p<0.05=weakly significant ,**p<0.01=significant ;†0.2<effect size (*ES*)<0.5=small effect,††0.5<*ES*<0.8=medium effect

Table S8. VOCs of significant difference between dwellings with detached and attached garages (median values, μg/m^3)

	Sample size	Ethylbenzene ***,††	2-Methylpentane***,††	Toluene **,†	n-Pentane***,††	Xylenes***,††	Cyclopentane **,†	Formaldehyde **,†	Benzene***,††	2-Methylbutane**,†	TVOC **,††
Detached	104	<LOD	<LOD	21	2.7	1.7	<LOD	13	<LOD	12	183
Attached	63	2.1	5.0	31	9.0	8.8	2.3	17	2.1	30	345

*p<0.05=weakly significant ,**p<0.01=significant ,***p<0.001=highly significant; †0.2<effect size (*ES*)<0.5=small effect,††0.5<*ES*<0.8=medium effect

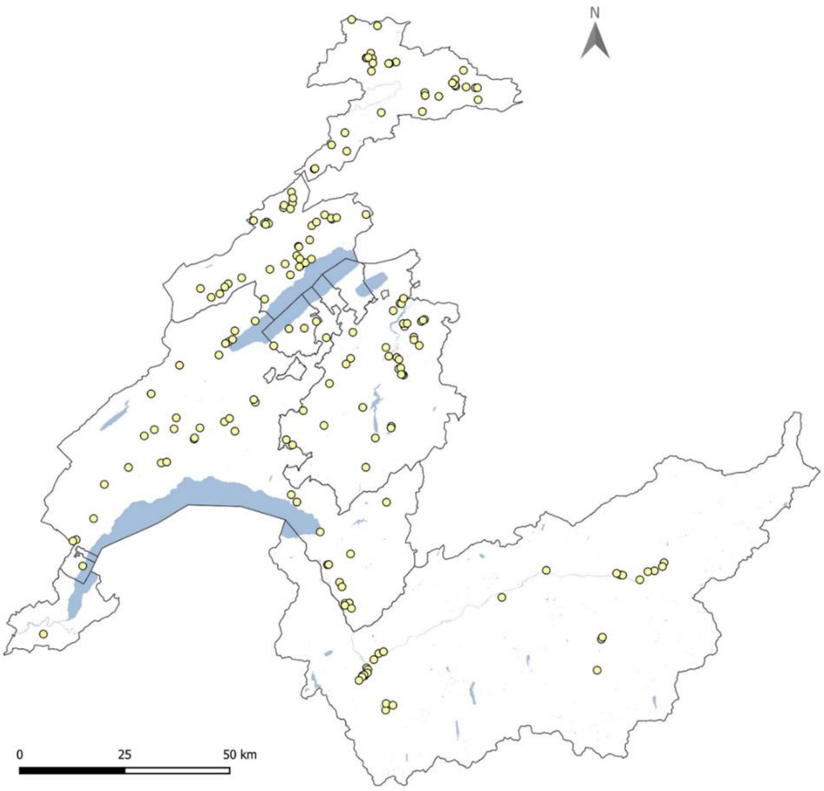

Figure S1. Locations of sampled dwellings in Switzerland

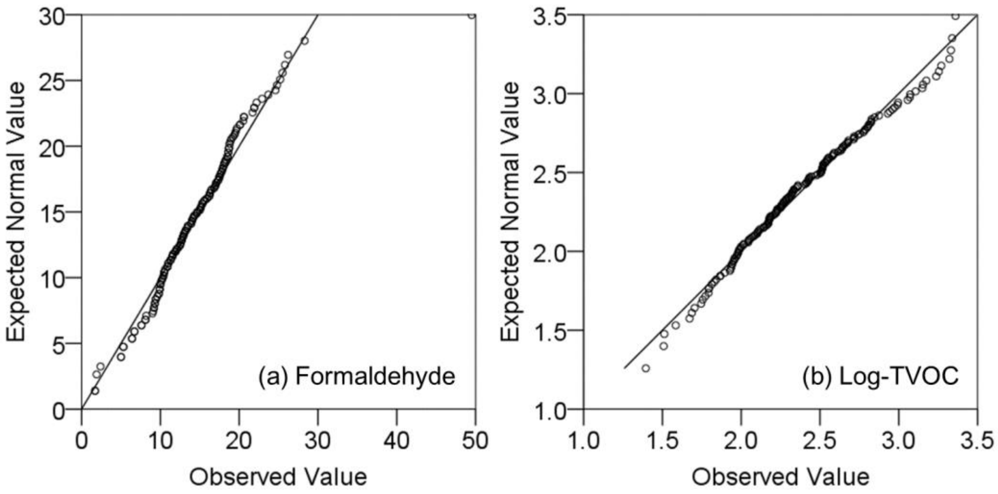

Figure S2. Q-Q plot of measured (a) formaldehyde and (b) log-transformed TVOC concentrations

Full Scan Organic Vapor Monitor

Cat. No. OV-00F

Description:
The sample is collected with a diffusive monitoring badge. There are no moving parts and no power is required. The air to be sampled diffuses through a porous perfluoropolymer membrane and is collected on a specially prepared carbon molecular sieve adsorbent. The badge is shipped to the sampling site and back to the analysis lab in a foil pouch that protects it from any gasses encountered during shipping. The returned sample is processed to screen over 150 compounds listed on page 4 -8. Advanced Chemical Sensors is accredited by American Industrial Hygiene Association (AIHA).

AIHA LAP, LLC
ACCREDITED LABORATORY
INDUSTRIAL HYGIENE
ISO/IEC 17025:2005
www.aihaaccreditedlabs.org
LAB #102047

Sampling Strategy:
The method used for sampling will be determined by the exact application and requires the judgment of an experienced industrial hygienists. For OSHA law enforcement, the badge should be worn on the collar of a worker's shirt for a full work shift (circa 8 h). Indoor air quality is easily measured by placing the badge at breathing level for 24 h. The exposure time should be chosen such that the concentrations of all chemicals of interest are above the reporting limits and the total mass collected is below circa 35 mg.

Shelf Life and Exposed Badge Holding Time:
The shelf life of a badge in an unopened pouch is 1 year. The badges should be analyzed within 2 weeks of exposure.

Reference Methods: OSHA Method 7 and EPA Method TO-15, modified for use with a diffusive sampler. ACS SOP II-62

Analyzer: Shimadzu GC-MS Model QP2010, AOC-20 auto sampler.

Analysis Procedures: The mass of each substance collected is measured by elution of the carbon with a known volume of solvent (carbon disulfide) and measurement of the concentration of each by capillary GC with a mass spectrometer detector (GC-MS). The mass of each substance is calculated from a unique calibration curve, one being generated for each substance (see Figure 1 for an example), and a recovery factor calculated from a media spike. The concentration (PPM) of each substance in the air sampled is calculated from this mass converted to volume of pure gas using the ideal gas law. The total volume of air that has passed through the membrane is determined from the time of use of the badge and a separate constant that describes the diffusion properties of the substance as well as its rate of capture by the sampler.

PPM = volume gas measured (mL) / total air volume tested (m^3)

The concentration of each substance detected and confirmed by the MS detector are calculated similarly and tabulated in a final report provided to the customer.

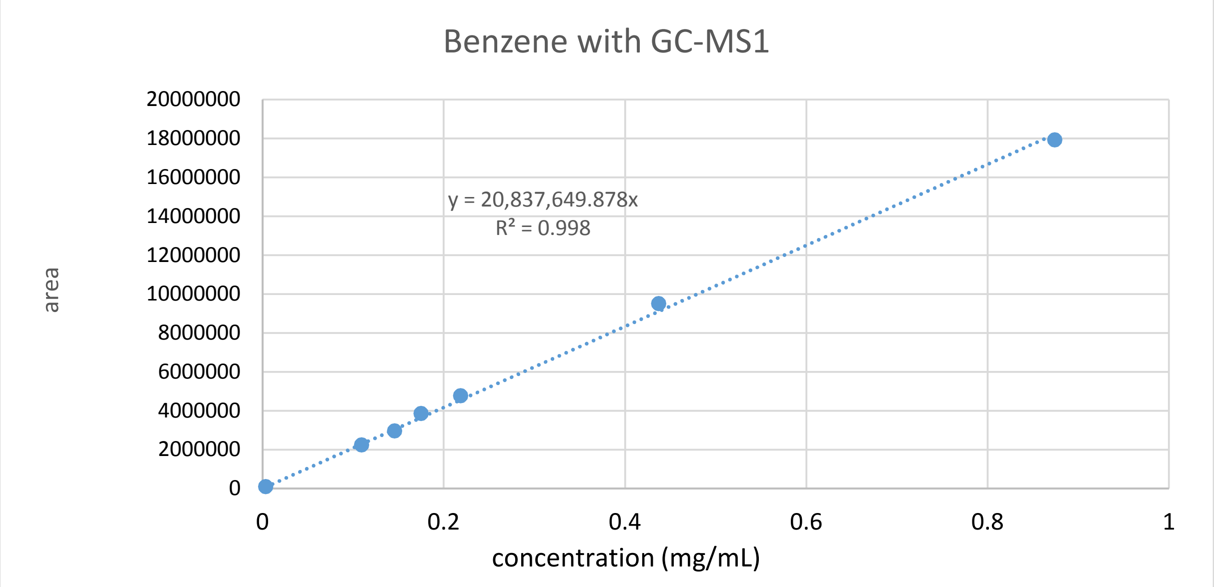

Figure 1. Plot of the detector response vs known mass of benzene. This regression equation is used to calculate the mass of benzene in customer.

Capacity: The carbon molecular sieve adsorbent has a maximum binding capacity of 25 mg of any single VOC on the list of substances measured, corresponding to >200 PPM exposure over 8 h period. For accurate measurements, we recommend that the mass of the sum of the VOCs measured not exceed 35 mg (calculated from the toluene calibration plot). ACS will advise the customer if this mass is exceeded.

Reporting and Detection Limit: The reporting limit of any substance is defined by the lowest concentration that can be measured with OSA < 25%, where

OSA % = bias % + 2*CV %

CV % = the coefficient of variation * 100 = (standard deviation / mean average)*100

bias % =((measurement - standard) / standard) * 100

The bias and SD are calculated from the analysis of 20 samples. This concentration is also used as the lowest calibrator. The reporting limits can always be lower than reported – the concentrations tested being determined by experience only. The detection limit is usually lower than the reporting limit, and is defined as being: 1) the lowest concentration that the MS detector can identify with >80% similarity to the standard MS and 2) a well-defined peak forms where the peak intensity is > 10 times the noise.

Accuracy: The accuracy (OSA) of the badge is always better than 25% when analyzed within the limits of the calibration plot established for each substance. If the area of the chromatogram is too high for the calibration plot, the sample is diluted and reanalyzed. If it is too low, it's reported as not detected unless the customer requests that it be reported to its detection limit. If the total mass present in the badge is found

to have exceeded 35 mg, we notify the customer and request resampling.

Reference Materials and Proficiency Testing Programs: We participate in AIHA's PAT (proficiency analytical testing) program for both solvents and diffusive badges, as well as an independent program administered by NSI Lab Solutions for perchloroethylene. We measure many different solvents when called for in the AIHA program, calibrating the GC-MS using authentic samples purchased from preferably the Aldrich Chemical Company. The similarity index function of the Shimadzu software is also used to ensure the identity of the reference materials by comparing its mass spectrum to a reference spectrum and providing a similarity number (>80% similarity necessary for acceptance). We have never failed to achieve less than an A rating in any proficiency test.

Interfering Factors: There are no known interfering factors. Blank samples are always run to confirm the background of the batch of badges being used for the test. For example, benzene has been found in blank adsorbent and the extraction solvent CS^2. Media and solvent blanks are always run to confirm that all substances measured are from the customer site. The shape of the GC peak is diagnostic of the purity of the eluting substance. Asymmetric peaks that differ from the calibrators noticeably are indicative of impurities with retention times very similar to benzene. The MS detector is also helpful in identifying co-eluting substances. Samples with asymmetric peaks will require reanalysis (for example, change in GC temperature or ramp) or resampling.

Effect of Environmental Conditions: The effect of humidity on the performance of the OV-00 badge has been explored and found to have no measurable effect in the range of 30%-92% RH. Increases in temperature from 24oC up to 37oC are found to have less than a 10% effect for each 10oC increase in the temperature. We don't recommend the correction of the data since the exact temperature effects on the collection of each chemical is not known and the correction factors that are known exactly are all within the overall error of the measurement.

	CAS #	Minimum Detection Limit (ppm)			Minimum Reporting Limit (ppm)		
		8 hrs	15 min	24 hrs	8 hrs	15 min	24 hrs
Acetoin	513-86-0	0.03	0.96	0.01	0.1	3.2	0.03
Acetone	67-64-1	0.02	0.64	0.01	0.1	3.2	0.03
Acetonitrile	75-05-8	0.03	0.96	0.01	0.1	3.2	0.03
Acetophenone	98-86-2	0.02	0.64	0.01	0.1	3.2	0.03
Acrylonitrile	107-13-1	0.03	0.96	0.01	0.05	1.6	0.02
Allyl Chloride	107-05-1	0.03	0.96	0.01	0.2	6.4	0.07
Alpha-Pinene (1R)	7785-70-8	0.01	0.32	0.01	0.05	1.6	0.02
Anethole	104-46-1	0.02	0.64	0.01	0.1	3.2	0.03
Benzene	71-43-2	0.01	0.32	0.01	0.05	1.6	0.02
Benzyl Acetate	140-11-4	0.03	0.96	0.01	0.1	3.2	0.03
Benzyl Alcohol	100-51-6	0.02	0.64	0.01	0.05	1.6	0.02
Benzyl Chloride	100-44-7	0.02	0.64	0.01	0.05	1.6	0.02
Beta-Citronellol	106-22-9	0.03	0.96	0.01	0.1	3.2	0.03
beta-Pinene	127-91-3	0.03	0.96	0.01	0.1	3.2	0.03
2-Bromoethanol	540-51-2	0.15	4.8	0.05	0.5	16	0.17
1,3-Butadiene	106-99-0	0.04	1.28	0.01	0.1	3.2	0.03
Butane	106-97-8	0.02	0.64	0.01	0.1	3.2	0.03
1-Butanethiol	109-79-5	0.05	1.6	0.02	0.2	6.4	0.07
2-Butanethiol	513-53-1	0.05	1.6	0.02	0.02	0.64	0.01
2-Butanone (MEK)	78-93-3	0.01	0.32	0.01	0.05	1.6	0.02
1-Butoxy-2-Propanol	5131-66-8	0.03	0.96	0.01	0.1	3.2	0.03
2-Butoxyethylacetate	112-07-2	0.03	0.96	0.01	0.1	3.2	0.03
Butyl Acetate	123-86-4	0.01	0.32	0.01	0.1	3.2	0.03
Butyl Acrylate	141-32-2	0.01	0.32	0.01	0.1	3.2	0.03
1-Butyl Alcohol	71-36-3	0.03	0.96	0.01	0.1	3.2	0.03
2-Butyl Alcohol	78-92-2	0.03	0.96	0.01	0.1	3.2	0.03
Butyl Ether	142-96-1	0.01	0.32	0.01	0.1	3.2	0.03
Butyl valerate	591-68-4	0.01	0.32	0.01	0.1	3.2	0.03
Butylcellosolve (2-Butoxyethanol)	111-76-2	0.02	0.64	0.01	0.05	1.6	0.02
Camphene	79-92-5	0.04	1.28	0.01	0.1	3.2	0.03
Camphor	76-22-2	0.03	0.96	0.01	0.1	3.2	0.03
Carbon Tetrachloride	56-23-5	0.05	1.6	0.02	0.1	3.2	0.03
3-Carene	13466-78-9	0.02	0.64	0.01	0.1	3.2	0.03
Carvone	99-49-0	0.02	0.64	0.01	0.1	3.2	0.03
Chlorobenzene	108-90-7	0.02	0.64	0.01	0.1	3.2	0.03
2-Chloroethanol	107-07-3	0.02	0.64	0.01	0.05	1.6	0.02
Chloroform	67-66-3	0.03	0.96	0.01	0.1	3.2	0.03
Chloromethane	74-87-3	0.06	1.92	0.02	0.2	6.4	0.07
1-chloropentane	543-59-9	0.04	1.28	0.01	0.1	3.2	0.03
Citral	5392-40-5	0.03	0.96	0.01	0.1	3.2	0.03

	CAS #	Minimum Detection Limit (ppm)			Minimum Reporting Limit (ppm)		
		8 hrs	15 min	24 hrs	8 hrs	15 min	24 hrs
Cumene	98-82-8	0.01	0.32	0.00	0.05	1.6	0.02
Cyclohexane	110-82-7	0.02	0.64	0.01	0.1	3.2	0.03
Cyclohexanol	108-93-0	0.03	0.96	0.01	0.1	3.2	0.03
Cyclohexanone	108-94-1	0.02	0.64	0.01	0.1	3.2	0.03
Cyclopentane	287-92-3	0.02	0.64	0.01	0.05	1.6	0.02
Cymene	99-87-6	0.01	0.32	0.00	0.05	1.6	0.02
Decamethylcyclopentasiloxane	141-63-9	0.01	0.32	0.00	0.05	1.6	0.02
Decanal	112-32-2	0.02	0.64	0.01	0.1	3.2	0.03
Decane	124-18-5	0.01	0.32	0.00	0.05	1.6	0.02
1-Decen	872-05-9	0.02	0.64	0.01	0.1	3.2	0.03
DEGDME	111-96-0	0.01	0.32	0.00	0.05	1.6	0.02
Diacetyl	431-03-8	0.02	0.64	0.01	0.05	1.6	0.02
1,2-Dichlorobenzene	95-50-1	0.02	0.64	0.01	0.1	3.2	0.03
1,3 Dichlorobenzene	541-73-1	0.02	0.64	0.01	0.1	3.2	0.03
1,4-Dichlorobenzene	106-46-7	0.02	0.64	0.01	0.1	3.2	0.03
1,1-Dichloroethane	75-34-3	0.02	0.64	0.01	0.05	1.6	0.02
1,2-Dichloroethane	107-06-2	0.03	0.96	0.01	0.1	3.2	0.03
Diethylene Glycol Ethyl Ether	111-90-0	0.04	1.28	0.01	0.2	6.4	0.07
Diethylene Glycol Monobutyl Ether	112-34-5	0.05	1.6	0.02	0.2	6.4	0.07
Diethylene Glycol Monobutyl Ether Acetate	124-17-4	0.05	1.6	0.02	0.2	6.4	0.07
Diisopropyl Ether	108-20-3	0.01	0.32	0.00	0.05	1.6	0.02
1,1-Dimethoxyethane	534-15-6	0.02	0.64	0.01	0.1	3.2	0.03
Dimethoxymethane	109-87-5	0.02	0.64	0.01	0.05	1.6	0.02
Dimethyl ether	115-10-6	0.02	0.64	0.01	0.1	3.2	0.03
1,2-Dimethylcyclohexane	583-57-3	0.02	0.64	0.01	0.1	3.2	0.03
cis-1,2-Dimethylcyclopentane	1192-18-3	0.02	0.64	0.01	0.1	3.2	0.03
1,2-Dimethylcyclopentane	2452-99-5	0.02	0.64	0.01	0.1	3.2	0.03
1,3-Dimethylcyclopentane	2453-00-1	0.02	0.64	0.01	0.1	3.2	0.03
2,4-Dimethylheptane	2213-23-2	0.02	0.64	0.01	0.05	1.6	0.02
2,5-Dimethylhexane	592-13-2	0.02	0.64	0.01	0.05	1.6	0.02
2,2-Dimethylpentane	590-35-2	0.02	0.64	0.01	0.05	1.6	0.02
2,3-Dimethylpentane	565-59-3	0.02	0.64	0.01	0.05	1.6	0.02
2,4-Dimethylpentane	108-08-7	0.02	0.64	0.01	0.05	1.6	0.02
3,3-Dimethylpentane	562-49-2	0.02	0.64	0.01	0.05	1.6	0.02
Dipropylene Glycol Methyl Ether	34590-94-8	0.03	0.96	0.01	0.1	3.2	0.03
Di-t-Butyl Peroxide	110-05-4	0.02	0.64	0.01	0.1	3.2	0.03
D-Limonene	5989-27-5	0.03	0.96	0.01	0.1	3.2	0.03
Dodecane	112-40-3	0.01	0.32	0.01	0.1	3.2	0.03
EGHE	112-25-4	0.02	0.64	0.01	0.1	3.2	0.03
Estragole (1,8-Cineole)	140-67-0	0.02	0.64	0.01	0.1	3.2	0.03

	CAS #	Minimum Detection Limit (ppm)			Minimum Reporting Limit (ppm)		
		8 hrs	15 min	24 hrs	8 hrs	15 min	24 hrs
ETBE (ethyl-tert-butyl-ether)	637-92-3	0.02	0.64	0.01	0.1	3.2	0.03
Ethanol	64-17-5	0.05	1.6	0.02	0.1	3.2	0.03
2-Ethoxyethanol	110-80-5	0.03	0.96	0.01	0.05	1.6	0.02
2-Ethoxyethylacetate	111-15-9	0.03	0.96	0.01	0.05	1.6	0.02
Ethyl 2-Methylbutanoate	7452-79-1	0.02	0.64	0.01	0.1	3.2	0.03
Ethyl 3-ethyoxypropionate	763-69-9	0.02	0.64	0.01	0.2	6.4	0.07
Ethyl Acetate	141-78-6	0.02	0.64	0.01	0.1	3.2	0.03
Ethyl Acrylate	140-88-5	0.02	0.64	0.01	0.05	1.6	0.02
Ethyl Benzene	100-41-4	0.01	0.32	0.01	0.1	3.2	0.03
Ethyl butyrate	105-54-4	0.03	0.96	0.01	0.1	3.2	0.03
Ethyl Ether	60-29-7	0.01	0.32	0.01	0.1	3.2	0.03
Ethyl Formate	109-94-4	0.03	0.96	0.01	0.1	3.2	0.03
Ethyl Isobutyrate	97-62-1	0.02	0.64	0.01	0.1	3.2	0.03
Ethyl Methacrylate	97-63-2	0.03	0.96	0.01	0.1	3.2	0.03
Ethyl Propionate	105-37-3	0.02	0.64	0.01	0.05	1.6	0.02
4-Ethyl Toluene	622-96-8	0.01	0.32	0.01	0.05	1.6	0.02
2-Ethyl-1-Hexanol	104-76-7	0.03	0.96	0.01	0.1	3.2	0.03
Ethylcyclopentane	1640-89-7	0.02	0.64	0.01	0.1	3.2	0.03
2-Ethyltoluene	611-14-3	0.01	0.32	0.01	0.05	1.6	0.02
Eucalyptol	470-82-6	0.03	0.96	0.01	0.1	3.2	0.03
Furfuraldehyde	98-01-1	0.02	0.64	0.01	0.1	3.2	0.03
Furfuryl Alcohol	98-00-0	0.08	2.56	0.03	0.2	6.4	0.07
Hexamethylcyclotrisiloxane	541-05-9	0.02	0.64	0.01	0.01	0.32	0.00
Hexamethyldisiloxane	107-46-0	0.01	0.32	0.01	0.05	1.6	0.02
Hexone (MIBK)	108-10-1	0.02	0.64	0.01	0.1	3.2	0.03
Isoamyl Acetate	123-92-2	0.02	0.64	0.01	0.05	1.6	0.02
Isobutane	75-28-5	0.02	0.64	0.01	0.05	1.6	0.02
Isobutyl Acetate	110-19-0	0.01	0.32	0.01	0.05	1.6	0.02
Isobutyl Isobutyrate	97-85-8	0.01	0.32	0.01	0.05	1.6	0.02
Isocineole	470-67-7	0.02	0.64	0.01	0.1	3.2	0.03
Isooctane	540-84-1	0.01	0.32	0.01	0.1	3.2	0.03
Isopropyl Acetate	108-21-4	0.02	0.64	0.01	0.05	1.6	0.02
Isopropyl Alcohol	67-63-0	0.02	0.64	0.01	0.1	3.2	0.03
Isopropyl chloride	75-29-6	0.03	0.96	0.01	0.1	3.2	0.03
Linalol	78-70-6	0.03	0.96	0.01	0.1	3.2	0.03
1-Methoxy-2-Propanol (PGME)	107-98-2	0.02	0.64	0.01	0.1	3.2	0.03
2-Methoxyethanol	109.86-4	0.03	0.96	0.01	0.1	3.2	0.03
Methyl Acetate	79-20-9	0.02	0.64	0.01	0.1	3.2	0.03
Methyl Acrylate	96-33-3	0.02	0.64	0.01	0.1	3.2	0.03
Methyl Bromide	74-83-9	0.03	0.96	0.01	0.05	1.6	0.02

	CAS #	Minimum Detection Limit (ppm)			Minimum Reporting Limit (ppm)		
		8 hrs	15 min	24 hrs	8 hrs	15 min	24 hrs
Methyl Butyrate	623-42-7	0.03	0.96	0.01	0.05	1.6	0.02
Methyl Chloroform	71-55-6	0.04	1.28	0.01	0.1	3.2	0.03
Methyl Formate	107-31-3	0.06	1.92	0.02	0.1	3.2	0.03
5-Methyl Furfural	620-02-0	0.03	0.96	0.01	0.1	3.2	0.03
Methyl Methacrylate	80-62-6	0.03	0.96	0.01	0.1	3.2	0.03
Methyl Styrene	98-83-9	0.02	0.64	0.01	0.05	1.6	0.02
3-Methyl-1-Butanol	123-51-3	0.03	0.96	0.01	0.1	3.2	0.03
2-Methyl-1-Butyl Acetate	624-41-9	0.02	0.64	0.01	0.05	1.6	0.02
2-Methyl-1-Propanol	78-83-1	0.03	0.96	0.01	0.1	3.2	0.03
2-methyl-1-propene	115-11-7	0.02	0.64	0.01	0.1	3.2	0.03
2-Methyl-2-Butanol	75-85-4	0.02	0.64	0.01	0.1	3.2	0.03
Methyl-2-methylbutyrate	868-57-5	0.02	0.64	0.01	0.1	3.2	0.03
2-Methyl-2-Propanol	75-65-0	0.02	0.64	0.01	0.1	3.2	0.03
1-Methyl-2-Pyrrolidinone	872-50-4	0.2	6.4	0.07	0.5	16	0.17
2-Methylbutane	78-78-4	0.02	0.64	0.01	0.05	1.6	0.02
Methylcyclohexane	108-87-2	0.02	0.64	0.01	0.05	1.6	0.02
Methylcyclopentane	96-37-7	0.02	0.64	0.01	0.05	1.6	0.02
Methylene Chloride	75-09-2	0.04	1.28	0.01	0.2	6.4	0.07
2-Methylheptane	592-27-8	0.02	0.64	0.01	0.05	1.6	0.02
2-Methylhexane	591-76-4	0.02	0.64	0.01	0.05	1.6	0.02
3-Methylhexane	589-34-4	0.02	0.64	0.01	0.05	1.6	0.02
3-Methyloctane	2216-33-3	0.02	0.64	0.01	0.05	1.6	0.02
2-Methylpentane	107-83-5	0.02	0.64	0.01	0.05	1.6	0.02
3-Methylpentane	96-14-0	0.02	0.64	0.01	0.05	1.6	0.02
Methyl-t-Butyl Ether	1634-04-4	0.02	0.64	0.01	0.05	1.6	0.02
2-Methyltetrahydrofuran	96-47-9	0.03	0.96	0.01	0.1	3.2	0.03
m-Ethyltoluene	620-14-4	0.03	0.96	0.01	0.1	3.2	0.03
m-Xylene	108-38-3	0.01	0.32	0.00	0.1	3.2	0.03
Naphtalene	91-20-3	0.03	0.96	0.01	0.05	1.6	0.02
n-Heptane	142-82-5	0.02	0.64	0.01	0.1	3.2	0.03
n-Hexane	110-54-3	0.02	0.64	0.01	0.1	3.2	0.03
n-Hexyl Acetate	142-92-7	0.03	0.96	0.01	0.1	3.2	0.03
Nonanal	124-19-6	0.03	0.96	0.01	0.1	3.2	0.03
Nonane	111-84-2	0.01	0.32	0.00	0.1	3.2	0.03
n-Pentane	109-66-0	0.02	0.64	0.01	0.1	3.2	0.03
n-Propyl Acetate	109-60-4	0.01	0.32	0.00	0.05	1.6	0.02
Octanal	124-13-0	0.03	0.96	0.01	0.1	3.2	0.03
Octane (all isomers)	111-65-9	0.01	0.32	0.00	0.05	1.6	0.02
1-Octene	111-66-0	0.03	0.96	0.01	0.1	3.2	0.03
o-Xylene	95-47-6	0.01	0.32	0.00	0.1	3.2	0.03

	CAS #	Minimum Detection Limit (ppm)			Minimum Reporting Limit (ppm)		
		8 hrs	15 min	24 hrs	8 hrs	15 min	24 hrs
2,3-Pentadione	600-14-6	0.03	0.96	0.01	0.1	3.2	0.03
2,2,4,6-Pentamethylheptane	31807-55-3	0.03	0.96	0.01	0.1	3.2	0.03
1-Pentanol	71-41-0	0.03	0.96	0.01	0.1	3.2	0.03
2-Pentanol	6032-29-7	0.03	0.96	0.01	0.1	3.2	0.03
2-Pentanone	107-87-9	0.01	0.32	0.00	0.05	1.6	0.02
2-Pentylfuran	3777-69-3	0.01	0.32	0.00	0.05	1.6	0.02
Perchloroethylene	127-18-4	0.03	0.96	0.01	0.05	1.6	0.02
4-Phenylcyclohexane	31017-40-0	0.05	1.6	0.02	0.2	6.4	0.07
Propyl Benzene	103-65-1	0.01	0.32	0.00	0.05	1.6	0.02
Propylene Glycol Methyl Ether Acetate	108-65-6	0.01	0.32	0.00	0.05	1.6	0.02
Pyridine	110-86-1	0.05	1.6	0.02	0.1	3.2	0.03
Styrene	100-42-5	0.02	0.64	0.01	0.3	9.6	0.10
Tetrahydrofuran	109-99-9	0.04	1.28	0.01	0.2	6.4	0.07
Thiophenol	108-98-5	0.05	1.6	0.02	0.1	3.2	0.03
Toluene	108-88-3	0.01	0.32	0.00	0.1	3.2	0.03
1,2,4-Trichlorobenzene	120-82-1	0.01	0.32	0.00	0.1	3.2	0.03
Trichloroethylene	79-01-6	0.04	1.28	0.01	0.1	3.2	0.03
Trimethyl Borate	121-43-7	0.03	0.96	0.01	0.1	3.2	0.03
1,2,3-Trimethylbenzene	526-73-8	0.01	0.32	0.00	0.1	3.2	0.03
1,2,4-Trimethylbenzene	95-63-6	0.01	0.32	0.00	0.1	3.2	0.03
1,3,5-Trimethylbenzène	108-67-8	0.01	0.32	0.00	0.1	3.2	0.03
Trimethylbenzene	25551-13-7	0.01	0.32	0.00	0.1	3.2	0.03
TVOC (eq toluene, on all pics)		0.03	0.96	0.01	0.1	3.2	0.03
Undecane	1120-21-4	0.01	0.32	0.00	0.1	3.2	0.03
Urethane	51-79-6	0.2	6.4	0.07	0.4	12.8	0.13
Vinyl Acetate	108-05-4	0.02	0.64	0.01	0.04	1.28	0.01
Xylenes	1330-20-7	0.01	0.32	0.00	0.1	3.2	0.03
